# Hypomorphic human *SEL1L* and *HRD1* variants uncouple multilayered ER-associated degradation machinery

**DOI:** 10.1172/JCI175448

**Published:** 2024-01-16

**Authors:** Katharine Umphred-Wilson, Stanley Adoro

**Affiliations:** Experimental Immunology Branch, National Cancer Institute, National Institutes of Health, Bethesda, Maryland, USA.

## Abstract

The suppressor of lin-12-like–HMG-CoA reductase degradation 1 (SEL1L-HRD1) complex of the endoplasmic reticulum–associated degradation (ERAD) machinery is a key cellular proteostasis pathway. Although previous studies have shown ERAD as promoting the development and maintenance of many cell types in mice, its importance to human physiology remained undetermined. In two articles in this issue of the *JCI*, Qi and colleagues describe four biallelic hypomorphic *SEL1L* and *HRD1* variants that were associated with neurodevelopment disorders, locomotor dysfunction, impaired immunity, and premature death in patients. These pathogenic SEL1L-HRD1 variants shine a light on the critical importance of ERAD in humans and pave the way for future studies dissecting ERAD mechanisms in specific cell types.

## ERAD pathway proteins

The endoplasmic reticulum (ER) is a major site of protein synthesis, folding, and maturation for membrane and secreted proteins. It is therefore imperative to have ER-intrinsic quality-control mechanisms that prevent accumulation of misfolded proteins in the ER lumen to prevent proteotoxic stress. The suppressor of lin-12-like–HMG-CoA reductase degradation 1 (SEL1L-HRD1) complex is the most conserved branch of the ER-associated degradation (ERAD) pathway, which senses and degrades misfolded proteins to maintain proteome homeostasis (termed “proteostasis”) (1). During ERAD, the molecular chaperones enhancing α-mannosidase like protein 1 (EDEM1), osteosarcoma amplified 9 (OS9), and ER lectin 1 (ERLEC1/XTP3B) bind to misfolded proteins in the ER lumen and bring them to SEL1L for degradation (1). Subsequent interaction of SEL1L with HRD1 facilitates the cytoplasmic export of substrates through its retrotranslocation channel and their ubiquitination by HRD1’s cytoplasmic tail (2). SEL1L and HRD1 also promote the stability of each other (3).

As germline deletion of *Sel1l* or *Hrd1* genes in mice results in embryonic or perinatal lethality (3, 4), conditional cell type–specific knockout models have been used to characterize the importance of these proteins and ERAD in animals. To date, critical requirements for SEL1L-HRD1 have been established in a wide range of cell types, including adipocytes, pancreatic β cells, and gut epithelial cells as well as in immune cells, notably hematopoietic stem cells (HSCs) and B and T cells (5–10). That defects in many of these cells and processes are incompatible with fetal and postnatal life provides one potential reason why pathogenic SEL1L-HRD1 variants have not been identified in humans. In the absence of any such natural variants, it was unclear how SEL1L-HRD1 and ERAD contributed to human physiology.

## *HRD1* and *SEL1L* pathogenic variants

**Figure 1:**
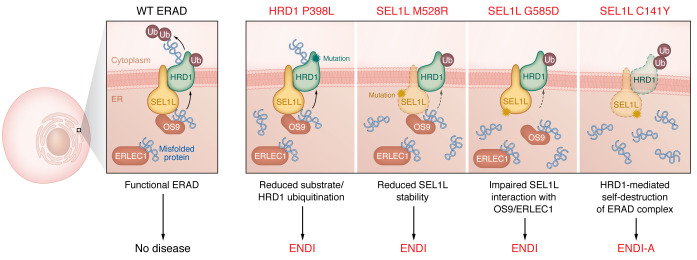
Human *SEL1L* and *HRD1* variants disrupt ERAD and cause disease. In normal cells, misfolded proteins in the ER are bound by the molecular chaperones ERLEC1 and OS9, both of which bring them to the SEL1L-HRD1 complex for ubiquitination and degradation to maintain cellular proteostasis. Qi and colleagues (11, 12) describe missense mutations in *SEL1L* and *HRD1* that encode SEL1L-HRD1 variants with hypomorphic activity in ERAD and were associated with a spectrum of developmental disorders termed ENDI and ENDI-A. These SEL1L and HRD1 variants appear to disrupt ERAD function by impairing either enzymatic activity (*HRD1* p.P398L), protein stability (*SEL1L* p.M528R and p.C141Y), or interactions with other ERAD components (*SEL1L* p.G585D).

In two complementary articles in this issue of the *JCI*, Qi and colleagues (11, 12) report the identification of one *HRD1* (p.P398L) and three *SEL1L* (p.G585D, p.M528R, and p.C141Y) pathogenic variants (Figure 1) arising from missense DNA mutations that altered amino acid residues within protein-protein interacting domains of SEL1L and HRD1. All four *SEL1L-HRD1* variants were biallelic mutations, suggesting that the presence of WT alleles might have protected against the development of any detectable phenotype in monoallelic carrier parents. Notably, children and adolescents in which the variants were identified presented with multiple signs of impaired neurodevelopment, severe hypotonia, and recurring infections. These SEL1L-HRD1 variants independently disrupted ERAD function and were associated with a spectrum of phenotypes that the investigators term ERAD-associated neurodevelopmental disorder with onset in infancy (ENDI) syndrome. Disease due to one of these variants (*SEL1L* p.C141Y), which was associated with a complete loss of B cells and antibodies, was termed ENDI-agammaglobulinemia (ENDI-A) syndrome.

In Wang et al. (11), Qi and colleagues describe *SEL1L-HRD1* variants in six patients from three unrelated consanguineous families from disparate geographical backgrounds (Italian, Saudi Arabian, and Moroccan). Whole-exome sequencing revealed that they all had biallelic mutations in the ERAD complex: *SEL1L* p.G585D, *SEL1L* p.M528R, and *HRD1* p.P398L. While all patients presented with dysmorphisms, developmental delays, intellectual disability, and short statures, only four of the six patients presented with seizures and microcephaly. Similarly, in Weis et al. (12), the authors report a *SEL1L* p.C141Y variant in five siblings from two consanguineous Slovakian families exhibiting ENDI symptoms along with frequent infections and premature death. Immunological tests failed to detect CD19^+^ lymphocytes or immunoglobulins in the blood of these patients, consistent with a loss of ERAD function in B cells as reported in mice (5). The basis of this variability in phenotype, despite similar impacts of these variants on ERAD activity, is unclear but suggests nuanced functions and interactions by SEL1L and HRD1. Importantly, that these *SEL1L* and *HRD1* variants were found in patients with seemingly different ancestry suggests a broad ethnic inheritance or acquisition of these mutations.

It is notable that all the *SEL1L* and *HRD1* variants were associated with neurodevelopmental disorders in patients given the critical importance of proteostasis and ERAD to normal brain and nervous system development (13–15). Mechanistically, ERAD dysfunction in the brain could result in the accumulation of misfolded proteins that cause proteotoxic stress, damaging neurons, and brain cells, which would then lead to impaired neuronal development and poor cognitive function. In line with this possibility, SEL1L silencing in neuronal stem cells caused the accumulation of autism spectrum disorder–related postsynaptic molecules CADM1 and Shank3 (14). Interestingly, this requirement for SEL1L-HRD1 in neurodevelopment appears to be shared across mammals, as the Qi group also recently described a *SEL1L* variant (p.S658P) in Finnish hounds that impaired SEL1L-HRD1 interaction and was associated with ENDI-like symptoms due to a loss of Purkinje neurons in the cerebellar cortex (16).

In comprehensive structure-function assays, Qi and coworkers elegantly demonstrate that these variants likely altered ERAD by disrupting the structure, stability, and interacting partners of both proteins (Figure 1). Specifically, the p.G585D variant, which is located in the substrate -binding domain of SEL1L, impaired SEL1L interaction with the chaperones OS9 and ERLEC1, thereby inhibiting substrate recruitment and degradation. The p.M528R, part of an α-helix in the SLR-M domain of SEL1L, resulted in reduced SEL1L stability, probably by impairing dimerization mediated by the SLR-M region or disrupting its interaction with HRD1. The C141Y mutation in SEL1L resulted in HRD1-mediated complex degradation due to a disruption in the disulfide bridge within the FNII domain. The only variant in HRD1, p.P398L, is located in the proline-rich region within the cytoplasmic tail that mediates its interaction with other ER membrane proteins (13) and resulted in a reduction in substrate ubiquitination and HRD1 autoubiquitination. Collectively, functional assays revealed that protein products encoded by the *SEL1L* and *HRD1* variants are hypomorphic, as they only partially impaired ERAD activity and were nonlethal, unlike germline murine *Sel1l* or *Hrd1* deficiency. Moreover, while all variants caused an increase in ERAD substrates, including IRE1α and CD147, they appeared to do so by different mechanisms. These observations suggest a multilayered role for SEL1L-HRD1 in the ERAD machinery in which distinct regions (represented by the variants) of either protein capture unique interacting partners to actuate different ERAD outputs. The hypomorphic activity of the variants may also explain the spectrum of phenotype and disease severity across patients with ENDI. Hypothetically, depending on cell type, the hypomorphic variants may detect and still provide some ERAD activity sufficient to mitigate low levels of proteotoxic stress.

## Conclusions and implications

By uncovering distinct mechanisms and essential roles for SEL1L and HRD1 variants, these exciting findings by Qi and colleagues raise intriguing new questions, answers to which should generate further insights into ERAD mechanisms that promote proteostasis. For example, impaired ERAD function is known to cause proteotoxic stress to cells due to an accumulation of misfolded proteins in the ER lumen, which activates the unfolded protein response (UPR) (17). Conceivably, chronic proteotoxic stress due to constitutive ERAD defect in developing neurons and nervous system tissues expressing SEL1L and HRD1 variants would cause UPR-induced apoptosis (17). However, despite ERAD defects and increased levels of misfolded proteins, there was no increase in UPR activity in cells expressing SEL1L or HRD1 variants. As this phenotype may be specific to fibroblasts or HEK293T cells in which the variants were analyzed, resolving exactly how the SEL1L and HRD1 variants cause ENDI-symptoms would require future studies modelling and examining the SEL1L-HRD1 crosstalk with other proteostasis pathways in relevant cell and tissue types. Additionally, SEL1L has been implicated as functioning outside of ERAD in regulating lipoprotein lipase secretion in adipocytes (18), suggesting that these SEL1L variants might regulate tissue development by potentially also modulating non–ERAD-related pathways.

In light of the reported roles for ERAD in the generation and maintenance of multiple hematopoietic cell lineages in mice (5–7), another intriguing issue is how these variants are dispensable for hematopoiesis and appear to only impair development of B cells in humans. Does this difference simply reflect the hypomorphic activity of the SEL1L and HRD1 variants, or does it indicate a mouse-versus-human nuance wherein human immune cells (compared with neurons) express distinct partners that are still able to interact with the SEL1L-HRD1 variants? Notably, none of the patients with ENDI showed any overt defects in hematopoiesis, unlike mice, in which SEL1L-deficient HSCs failed to efficiently generate blood cells (6, 9). In individuals with ENDI-A with the *SEL1L* p.C141Y variant, only B cells (and consequently antibodies) were deficient, and these patients also lacked detectable COVID-specific memory T cells after COVID infection (12). As these memory defects could be due to a lack of CD4^+^ T cell help, it would be worth investigating specific functions of T cells harboring these variants given reported roles for SEL1L and HRD1 in T cell development and function (7, 19). Moreover, while T cell numbers were normal in patients with ENDI-A, their skewed CD4/CD8 ratios (normal: 1.5–2.5; ENDI-A: 0.77–0.83) suggest defects in T cell homeostasis.

In conclusion, these reports from Qi and colleagues establish the importance of ERAD in humans and set the stage for precise dissection of ERAD and its crosstalk with other pathways that promote proteostasis. Their findings establish ENDI and ENDI-A as clinically relevant neurodegenerative disorders that may be corrected by reversal of the mutant genes. Importantly, by modeling these variants in cell lines and animal models, future studies can now uncouple SEL1L-HRD1 interactions from substrate degradation to better understand nuances in ERAD function.

## Version 1 01/16/2024

Electronic publication
